# Secondary Syphilis

**DOI:** 10.1155/S106474499700046X

**Published:** 1997

**Authors:** David E. Soper

**Affiliations:** Department of Obstetrics and Gynecology Medical University of South Carolina 171 Ashley Avenue Charleston SC 29401 USA


					Infectious Diseases in Obstetrics and Gynecology 5:272 (I 997)
(C) 1998 Wiley-Liss, Inc.
Images in Infectious Diseases in Obstetrics and Gynecology
Section Editor: David E. Soper, M.D.
Secondary Syphilis
David E. Soper, MD
Department of Obstetrics and Gynecology, Medical University
ofSouth Carolina, Charleston, SC 29401
LEGEND TO IMAGE
A 27-year-old black woman presented with complaints of
skin lesions on the vulva, back, nipple and ear. Darkfield
examination of the cxudate from the moist vulvar pap-
ules (A) revealed motile spirochetes. A VDRL was re-
active at a titer of 1:1,024. A papular lesion with raised
borders (annular syphilid) (B) was discovered during ex-
amination of the back. Split papules were also discovered
on the nipple (C) and right ear (D). A single intramus-
cular injection .of benzathine penicillin G (2.4 million
units) was curative. (C) 1998 Wiley-Liss, Inc.
Images in Infectious Diseases in Obstetrics and Gynecology presents clinically important visual images that a practitioner in women's health
might encounter. If you have a high-quality color or black-and-white photograph of slide representing such an image that you would
like considered for publication, send it with a descriptive legend to David E. Soper, MD, Medical University of South Carolina, 171
Ashley Avenue, Charleston, SC 29401-2233.
Images is made possible through an educational grant from Pfizer, Inc.

				

